# Brassinosteroids induced drought resistance of contrasting drought-responsive genotypes of maize at physiological and transcriptomic levels

**DOI:** 10.3389/fpls.2022.961680

**Published:** 2022-10-25

**Authors:** Syed Faheem Anjum Gillani, Zelong Zhuang, Adnan Rasheed, Inzamam Ul Haq, Asim Abbasi, Shakil Ahmed, Yinxia Wang, Muhammad Tajammal Khan, Rehana Sardar, Yunling Peng

**Affiliations:** ^1^ Gansu Provincial Key Lab of Arid Land Crop Science, College of Agronomy, Lanzhou, China; ^2^ College of Agronomy, Hunan Agricultural University, Changsha, China; ^3^ Crop Breeding Department, Jilin Changfa Modern Agricultural Science and Technology Group, co., Ltd., Changchun, China; ^4^ College of Plant Protection, Gansu Agricultural University, Lanzhou, China; ^5^ Department of Environmental Sciences, Kohsar University, Murree, Pakistan; ^6^ Institute of Botany, University of the Punjab, Lahore, Pakistan; ^7^ Department of Botany, Division of Science and Technology, University of Education, Lahore, Pakistan

**Keywords:** GO analysis, KEGG, metabolism, transcriptome, zea mays

## Abstract

The present study investigated the brassinosteroid-induced drought resistance of contrasting drought-responsive maize genotypes at physiological and transcriptomic levels. The brassinosteroid (BR) contents along with different morphology characteristics, viz., plant height (PH), shoot dry weight (SDW), root dry weight (RDW), number of leaves (NL), the specific mass of the fourth leaf, and antioxidant activities, were investigated in two maize lines that differed in their degree of drought tolerance. In response to either control, drought, or brassinosteroid treatments, the KEGG enrichment analysis showed that plant hormonal signal transduction and starch and sucrose metabolism were augmented in both lines. In contrast, the phenylpropanoid biosynthesis was augmented in lines H21L0R1 and 478. Our results demonstrate drought-responsive molecular mechanisms and provide valuable information regarding candidate gene resources for drought improvement in maize crop. The differences observed for BR content among the maize lines were correlated with their degree of drought tolerance, as the highly tolerant genotype showed higher BR content under drought stress.

## Introduction

Maize (*Zea mays* L.), the most significant grain crop on the earth, is generally cultivated for food, feed, and biofuel production (Ansari et al., 2022). Climatic changes have significantly affected crop growth and yield in recent times (Fàbregas et al., 2018; Singhal et al., 2022). Moreover, different climate-induced abiotic stresses severely affect the growth and yield of major crops worldwide.

Among these abiotic constraints, drought is of prime importance because of its rapidly increasing severity in the coming future (Chen Y. et al., 2019). Drought stress inhibits maize seedling growth and crop development and reduces yield, particularly at the grain filling stage. However, yield losses caused by water scarcity in maize may vary depending on drought severity and the phenological stage of the crop (Hu et al., 2018). In this regard, plant growth-promoting hormones play a key role in safeguarding maize growth under drought stress, as evidenced by an earlier study, which showed that ABA levels speed up the kernel filling process (Jin et al., 2019).

The maize plant has a simple structure, and its unique germplasm makes it a more valuable crop than other cereals (Corso et al., 2015). Although significant progress has been made in investigating maize response to drought stress, previous studies have focused on physiological and metabolic or single-trait responses (Estrella-Maldonado et al., 2021). The high-throughput sequencing frameworks that rely on the RNA level and their analysis results have profoundly changed our viewpoint on the extent and heterogeneity of the maize transcriptome to a substantial degree over the last decade (Faghani et al., 2015). Whatever the case, most transcriptomic studies focused on the vegetative stage of the tested crop plants. Our perception of maize drought stress response structures and attributes highlights the need for further studies (Kosová et al., 2016).

Plants experience various environmental challenges during their life cycle that impact their growth and development (Anjum et al., 2017a). Drought is one of the most detrimental environmental conditions, eventually lowering agricultural yields (Anjum et al., 2016). During the vegetative phase, drought stress can limit growth rate, extend the vegetative development stage, and change the distribution of carbohydrates in maize (Aleem et al., 2021).

It has been demonstrated that short-duration water deficiencies result in 28%–32% losses in dry weight 66%–93% during the tasseling and ear formation stages of the maize crop, respectively. The number of kernel rows is established at the V9 stage when several ear shoots appear (Fracasso et al., 2016). The maize plant starts accumulating nutrients and dry weight quickly and steadily at the V10 stage and keeps doing so throughout the reproductive period (Hao et al., 2020). Long-term dryness during the pre-flowering stage has also been demonstrated to decrease the ultimate size of certain leaves and internodes, postpone the development of tassels and silk, and result in 15% to 20% yield losses (Haider et al., 2017).

Different agronomic and physiological techniques are used to reduce the negative effects of drought and to help plants develop a resistance to it (Janiak et al., 2018). The use of plant growth regulators is one of the potential methods to increase agricultural output under difficult conditions. Exogenously administered growth regulators have been shown to enhance plants’ ability to withstand a variety of abiotic stressors, including drought and heavy metal salt pressure (Tanveer et al., 2019). Brassinosteroids (BRs) serve various crucial functions in plant growth and belong to a novel family of phytohormones and development. On the steroidal side, each of these BRs has a 22R, 23R-diol structural characteristic. In light of this, molecules with 22R and 23R hydroxyls are much more active than their synthetic equivalents with 22S and 23S hydroxyls. In contrast, BR members with a 24S-methyl or -ethyl group exhibit 10 times more bioactivity in the native 22R, 23R configuration than do hormones with a 24R-alkyl function, highlighting the importance of the stereochemistry at this asymmetric center (Kang et al., 2019).

The steroidal phytohormones known as brassinosteroids (BRs) control the growth and development of plants. While the network-like pathways for BR production in *Arabidopsis* have been well characterized, it is still unclear what functions some biosynthetic enzymes play in the various sub-pathways (Kumar et al., 2018). Broad substrate specificities of the involved enzymes enable metabolite flow across a number of routes during BR production, which takes place along a metabolic grid. In the plant kingdom, three substances—cholesterol, sitosterol, and campesterol—are used to create BLs. The two sterols most prevalent in plant membranes are sitosterol and campesterol; however, there have been two methods for the biosynthesis of brassinolides that start with campesterol (Tanveer et al., 2018).

Similarly, around the time of pollination, 5 days of drought stress causes abnormal embryo development and a significant reduction in kernel number. The maize ear leaf makes a significant contribution to the buildup of biomass as a result of photosynthesis (Liu et al., 2019). Five or six leaves close to and above the ear generate most of the photosynthate for kernel production. The size of the “source” in plants is reduced due to drought stress, which causes a sharp reduction in the photosynthetic rate. Plants at the seedling stage were primarily used to survey and describe gene regulatory networks of the drought stress response (Wang et al., 2014).

Different plants face osmotic and drought stress, which leads to the expression of many genes. They may generally be separated into ABA-dependent and other signaling pathways. Since it affects stomatal closure and the production of stress-responsive genes, ABA is a key phytohormone in the drought stress response in plants. Hormones play a key role in plant response to abiotic stresses; osmotic stress signaling, the calcium-dependent route, mitogen-activated kinase-mediated signaling, phospholipid signaling, and reactive oxygen species (ROS) signaling are some other signaling mechanisms for drought stress tolerance (Anjum et al., 2017b).

In national plants, maize cross varieties are often preferred over homozygous regular lines since they are the most heterosis-rich crop in modern agricultural production. Separated from the normal line, the cross-variety plant is more rooted (Luo et al., 2018). The improvement steps are not performed in comparable field circumstances. There were not as many features passed on to the cultivars by the drought treatment as there would have been under normal conditions. Samples showed enormous rates of vegetation when exposed to the stress of the drought. Like all other samples, these exhibited the most pronounced leaves (Li et al., 2017).

While the more established leaves of different plants exposed to dry solid season areas showed signs of aging (the lower two leaves were dry or yellow, and the third leaf also started to senesce), this did not significantly affect other samples, which commonly developed under stress conditions (just toning down) (Liu et al., 2021). Our findings have improved the translation of maize drought response instruments and provided a theoretical foundation for breeding novel maize cultivars resistant to dry periods (Singh et al., 2017).

As normal biological changes occur, the significant financial difficulties caused by drought in agricultural productivity will get worse, and the financial difficulties caused by droughts in agricultural productivity will worsen (Dossa et al., 2017). Several cutting-edge technologies are being used and exploited to forecast these shifts (Mishra et al., 2022). It has been recommended to use polyethylene glycol (PEG), amino acids, cell protectors, phytohormones, minerals, typical eccentric treatments, and so on (Muthusamy et al., 2016). Brassinosteroids are a group of steroidal phytohormones included in combinations (BR). Due to their non-toxicity, non-mutagenicity, and eco-friendly nature, as well as their effectiveness at low concentrations, ease of use, and potential for deceptive mixing on a commercial scale, the BRs are a good choice for agricultural use (Zhao et al., 2020). Drought tolerance is a complex polygenic trait and is still not fully understood. This study aimed to investigate the drought tolerance of two contrasting maize lines. We performed the transcriptome analysis to identify the different genes controlling drought tolerance in maize and also checked the role of BRs in mitigating the drought-induced changes in plants.

## Material and methods

### Plant material, experimental design, and BR treatment

Seeds of two maize inbred lines (ILs), drought-sensitive (H21) and drought-tolerant (478), were provided by the Gansu Provincial Key Lab of Arid Land Crop Science, College of Agronomy, Gansu Agricultural University, Lanzhou, China, and were sown in pots. Two inbred lines were chosen to identify the tolerant genes and to check the Br role in drought tolerance. Br hormones also improve the plant growth in maize inbred lines; this showed that Br hormones could improve drought tolerance in sensitive lines, and tolerant genes identified in tolerant inbred lines can be used in molecular breeding. The pots were placed in a temperature-controlled environment in the laboratory (temperature 25 ± 5; relative humidity 65 ± 5; light/dark: 16/8 h). Up to 10 days following emergence, all the plants received enough water to maintain a steady moisture level; at that point, the application of treatments started. Treatments consisted of two levels of drought stress (20% and 0.3%) and three levels of BR (0, 25, and 50 ml). Fifty milliliters of 20% PEG 6000 was applied every 3 days to induce drought, and distilled water was applied in control. Seedlings at the four fully developed leaf stages were selected to determine morphological and physiological parameters. For transcriptome analysis, samples were stored at -80°C in liquid nitrogen. The experiment was carried out under a completely randomized design (CRD) with three replications. The BRs (including castasterone, 24-epicastasterone, brassinolide, and 24-epibrassinolide) have been extracted with ice-cold 80% aqueous methanol solution. The stock solutions of BRs were prepared at a concentration of 100 mg/l in methanol and stored at -18°C in the dark. Standards of lower concentrations were prepared weekly by the serial dilution of the stock solution with methanol and were stored at 4°C in the dark. During this analysis, a number of physiological and morphological parameters were analyzed, some of which involved growth parameters, for example, plant height, root dry weight, shoot dry weight, the number of leaves, leaf area, the specific mass of the fourth leaf, antioxidant activity, and malondialdehyde and chlorophyll contents.

### Transcriptomic analysis

#### RNA extraction library construction and sequencing

The outright RNA was removed using a TRIzol reagent (Thermo Fisher, 15596018). The total amount of RNA and its quality were determined using the Bioanalyzer 2100 and the RNA 6000 Nano LabChip Kit (Agilent, California, United States of America, 5067-1511), and first-rate RNA tests with RIN numbers greater than 7.0 were used in the construction of the sequencing library. After the total RNA had been removed, the mRNA remaining in the pure RNA (5 μg) was cleaned using two rounds of filtering with Dynabeads Oligo (dT) (Thermo Fisher in California, USA). After being filtered, the mRNA was fragmented into shorter portions by utilizing divalent cations at a higher temperature (using the Magnesium RNA Fragmentation Module from NEB, cat. e6150, USA) for 5–7 min at 94°C. After that, the cut RNA pieces were inversely unraveled to make the cDNA using SuperScript™ II Reverse Transcriptase (Invitrogen, cat. 1896649, USA). The cDNA was then utilized to incorporate U-named second-deserted DNAs with *E. coli* DNA polymerase I (NEB, cat.m0209, USA) and RNase H (NEB, cat.m0297, USA) and dUTP Solution (Thermo Fisher, cat. R0133, USA). The unpolished terminations of each strand were given an A-base coating, which prepared the strands to be ligated to the recorded connectors.

Each connector had a T-base shade built into it to ligate the connection to the A-followed partitioned DNA. After affixing twofold document connections to the parts, AM Pure XP dabs were used to carry out size verification. After the power labile UD Genzyme (NEB, cat.m0280, USA) treatment of the U-named second-deserted DNAs, the ligated things were escalated with PCR using the following conditions: starting denaturation at 95°C for 3 min; eight examples of denaturation at 98°C for 15 s, treating at 60°C for 15 s, and increase at 72°C for 30 s; and a short-time later last extension at 72°C for 5 min. The preceding cDNA libraries had expansion sizes that ranged from 300 to 50 bp on average. Following the manufacturer’s instruction, we completed the 2 × 150 bp matched end sequencing (PE150) using an Illumina NovaSeq™ 6000 (LC-Bio Technology Co., Ltd., Hangzhou, China).

#### Sequence and filtering of clean reads

A cDNA library constructed by invention using the pooled RNA from the tests done with the Illumina NovaSeq™ 6000 succession stage was sequenced. We sequenced the transcriptome using the Illumina paired-end RNA-seq approach; reads obtained from the sequencing machines include raw reads containing adapters or low-quality bases, affecting the following assembly and analysis (Martin, 2011). Thus, to get high-quality clean reads, reads were further filtered by Cutadapt. The parameters were as follows: 1) removing reads containing adapters; 2) removing reads containing polyA and poly; 3) removing reads containing more than 5% of unknown nucleotides (N); 4) removing low-quality reads containing more than 20% of low-quality (Q-value ≤ 20) bases. After that, the quality of the sequence was checked using FastQC. It was observed that Q20, Q30, and GC content of the data had not been contaminated. Following that, a total of gigabase pairs worth of reads with clean ends was generated (Thompson et al., 2020). The raw sequencing data have been submitted to the NCBI Gene Expression Omnibus (GEO) databases or the NCBI Short Read Archive (SRA) with accession numbers. Both of these archives have been given accession numbers.

### Pathway enrichment analysis (KEGG)

Qualities generally cooperate in assuming parts in specific organic capacities. Pathway-based investigation assists with understanding the rates of different physical processes. KEGG is a significant public pathway-related information base. Pathway improvement examination distinguished enhanced metabolic pathways or sign transduction pathways in DEGs in contrast with the entire genome foundation. Pathway advancement examination utilized Omic Share to determine extraordinarily upgraded metabolic pathways or sign transduction pathways in differentially communicated qualities. A hypergeometric test-taking FDR characterized extensively improved pathways in differentially communicated qualities (Aleem et al., 2021).

### RT-PCR

The PCR assays were performed on the Rotor-Gene 3000 detection system (Corbett Research, Singapore) using One-Step PrimeScript™ RT-PCR Kit (Perfect Real Time) (Takara). All sets of reactions were carried out in a final volume of 20 l, each containing 10 l of 2× One-Step RT-PCR Buffer III, 0.4 l of MCMVf (10 M), 0.4 l of MCMVr (10 M), 0.8 l of probe (10 M), 0.4 l of Ex Taq™ (Takara) HS (5 U/l), 0.4 l of PrimeScript™ RT Enzyme Mix II, 1.0 l of total RNA or 1.0 l of RNA transcripts, and 6.6 l of RNase-free dH_2_O. Amplification reactions were performed as follows: 42°C for 5 min; 95°C for 10 s; 40 cycles of 95°C for 5 s, and 60°C for 20 s. The specificity of this TaqMan assay was evaluated using six different reactions, including water control. Using the TaqMan probe, strong fluorescent signals were detected only from reactions with samples, while the signals from four other samples along with the water control were superposed to the baseline under optimized reaction conditions. These samples can be differentiated from the four other maize samples by comparing the signals of different levels. The PCR products were analyzed further by agarose gel electrophoresis. The assay with the sample displayed the expected band of 67 bp and an unexpected faint band above the 67-bp band, whereas those of the four others did not.

### Statistical analysis

Statistical significances between control and drought treatment were tested using the Newman–Keuls method at P < 0.05 by IBM^®^ SPSS^®^, and data were generated by SigmaPlot 12.5. PCA was performed with the help of XLSTAT statistical software.

## Results

### Morphological and physiological traits of maize cultivars

During this analysis, a number of physiological and morphological parameters were analyzed, some of which involved growth parameters, for example, plant height, root dry weight, number of leaves, leaf area, and number of stems. Similarly, as far as functional parameters are concerned, they involve photosynthetic activity, leaf hydration, chlorophyll content, carbon dioxide diffusion, and CAT and pigment content. Phenotypic diversity for all traits was found under control and drought. Chlorophyll content and dry load were reduced by 90% during the drought, but a 40% reduction was seen in plant height. The two maize cultivars have been growing under identical circumstances, except for certain drought treatments. In both cultivars, as shown in the figure, there is a noticeable difference in plant height under two treatments; plants in drought treatment are shorter than those in water treatment. Both cultivars of drought-treated plants displayed withered leaves. However, under the two treatments, there was no visible variation in the shape of the ears between the two cultivars. When drought stress was applied to the samples under observation, they showed visible variations. According to the data obtained after analysis, root dry weight showed rough and symmetric results under the two conditions. Exceptions of plant height and any other variables’ phenotypic advantages were obtained in two situations. A couple of layouts look at each boundary where H21 displayed a more pronounced plant height. Additionally, while talking about root dry weight, 478 performs better than H21. A comparison of results for all boundaries is shown in **Tables 1**, **2**.

**Table 1:** Advanced test results of ANOVA.

Source	SS	df	MS	
Between-treatments	0	1	0	F = 0.01776
Within-treatments	0.0302	30	0.001	
Total	0.0302	31		

**Table 2:** ANOVA test result.

Sr. #	Treatments			
**1**	1	2	3	Total
**2**	N	16	16	32
**3**	∑X	0.3903	0.3664	0.7566
**4**	Mean	0.0244	0.0229	0.024
**5**	∑X^2^	0.0248	0.0233	0.0481
**6**	Std. dev.	0.0319	0.0315	0.0312

### Pathway enrichment analysis (KEGG)

**Figure 1:**
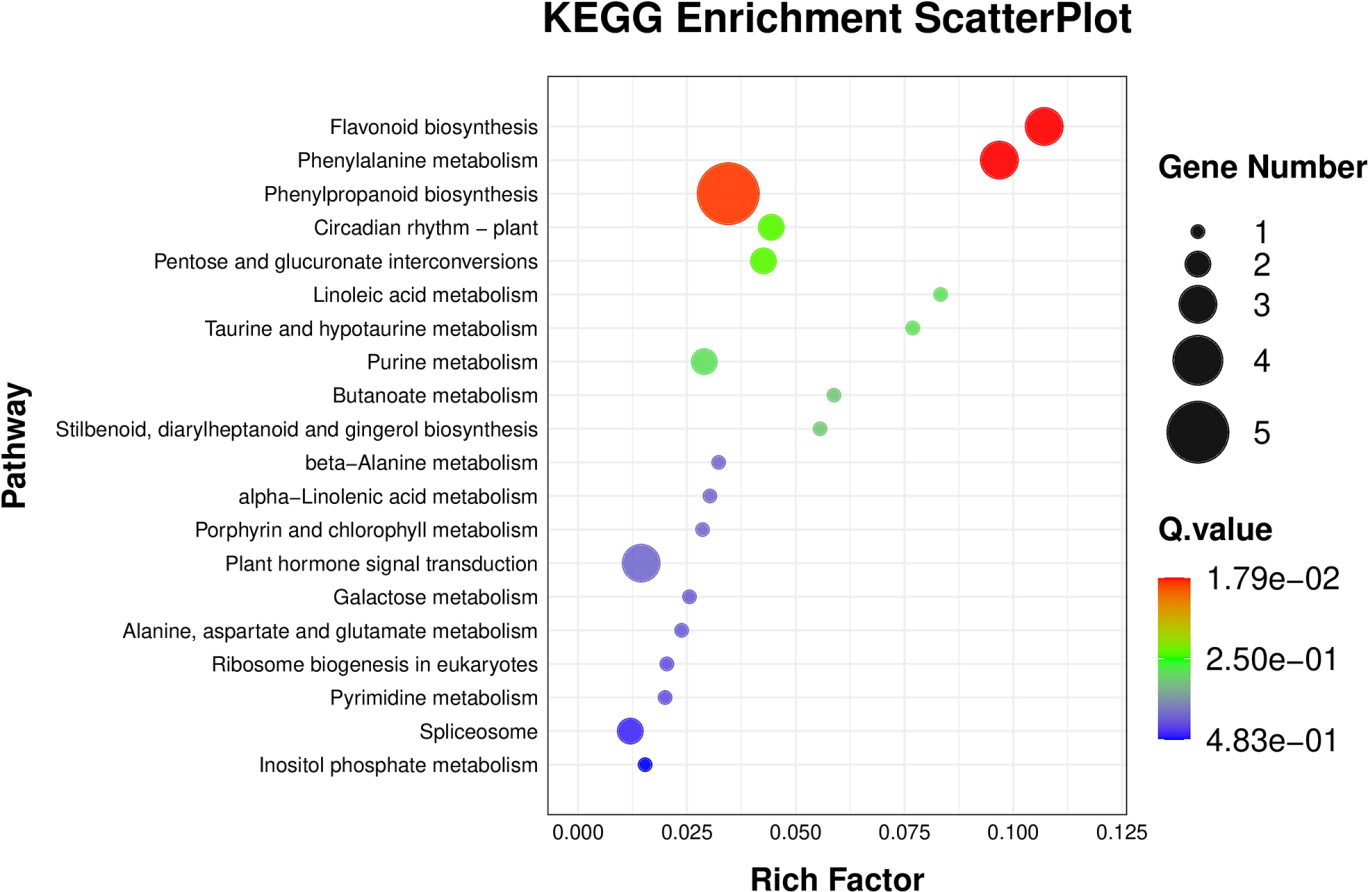
KEGG pathway enrichment scatter plot. The color of the point represents the size of the q-value. The smaller the q-value, the closer the point color to the red color. The point size expresses the number of differential genes in each pathway.

**Figure 2:**
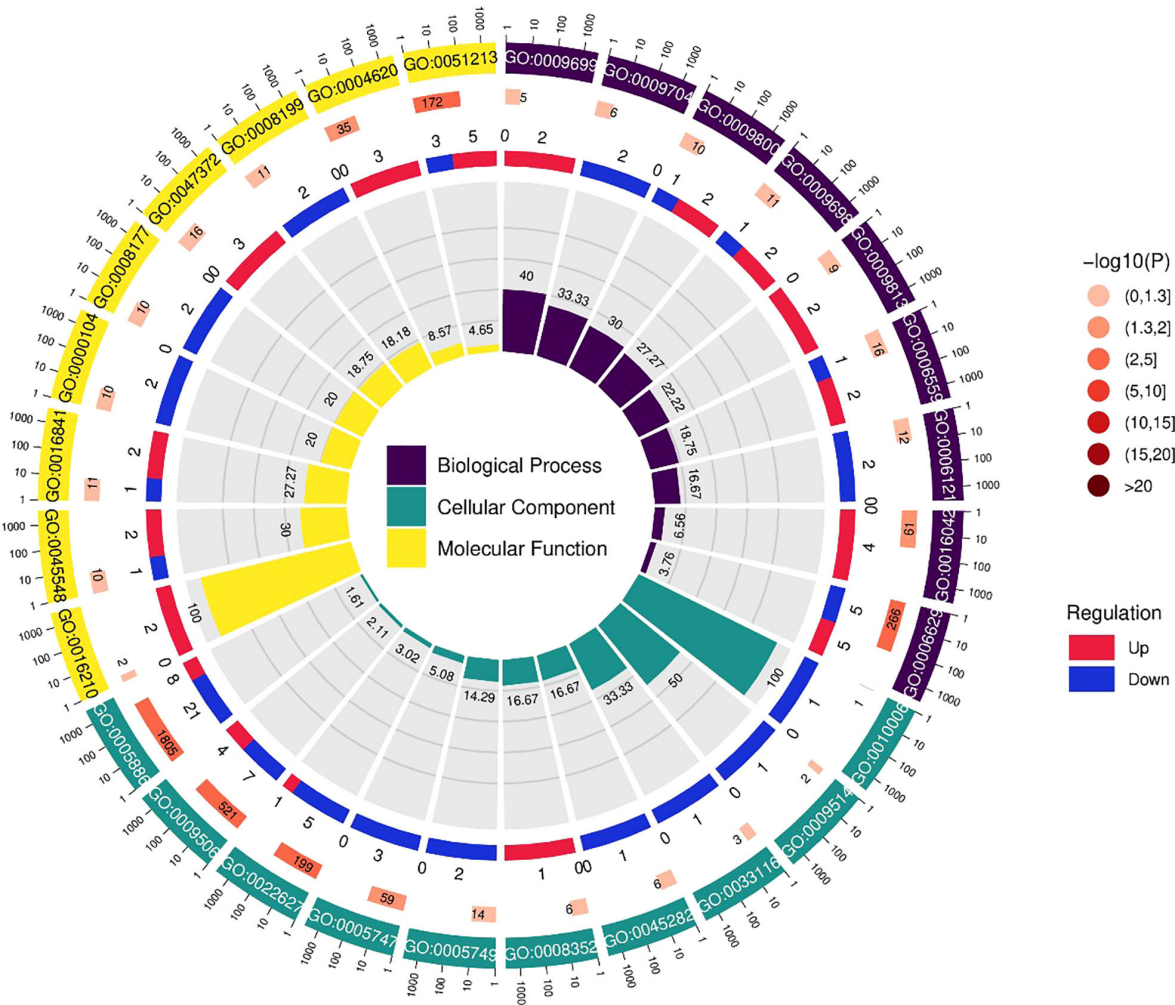
KEGG enrichment axial plot shows the DEG enrichment analysis results in the KEGG pathway.

**Figure 3:**
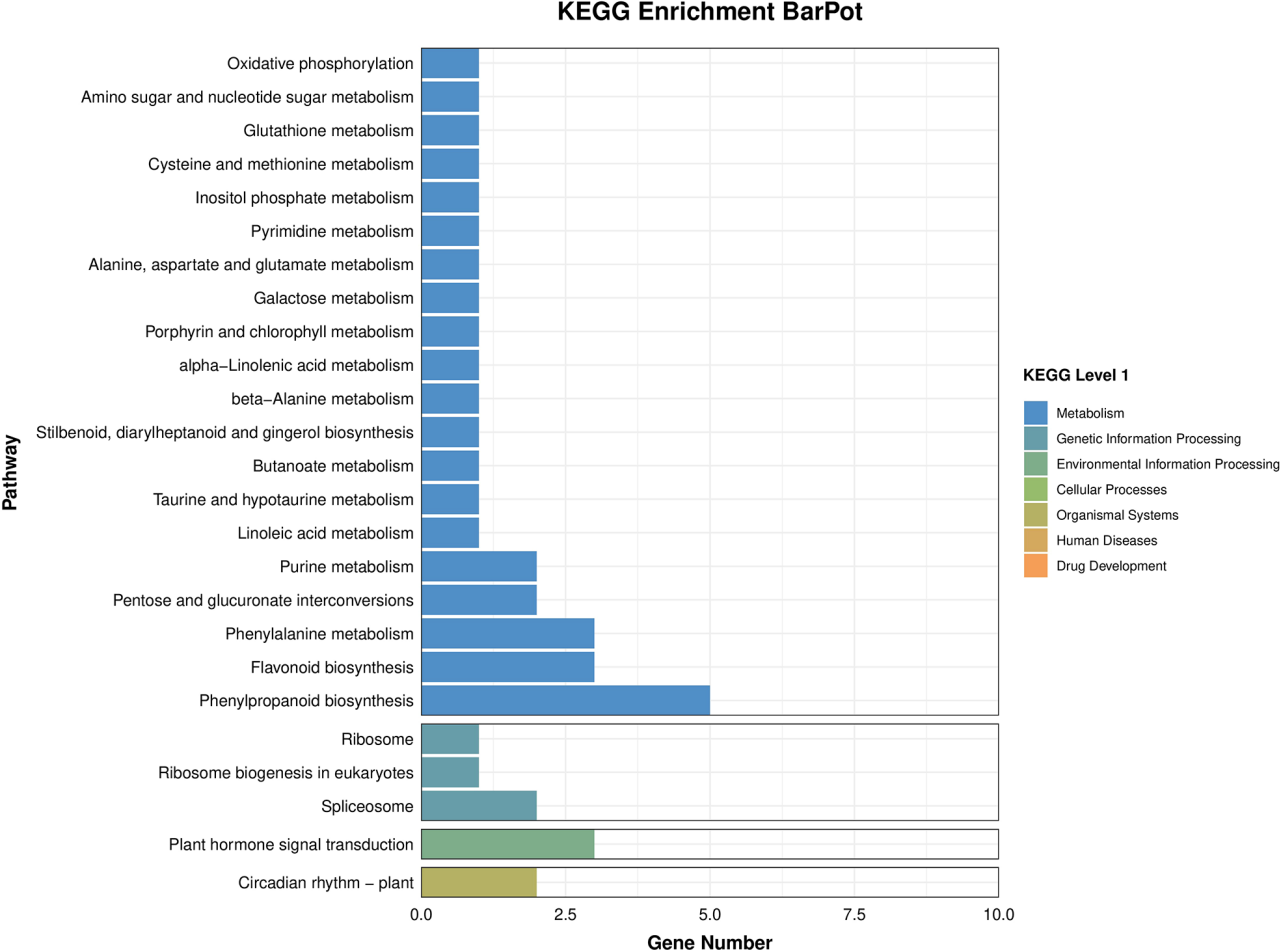
Classification of differentially expressed genes. Total gene counts of KEEG terms are associated with all genes.

**Figure 4:**
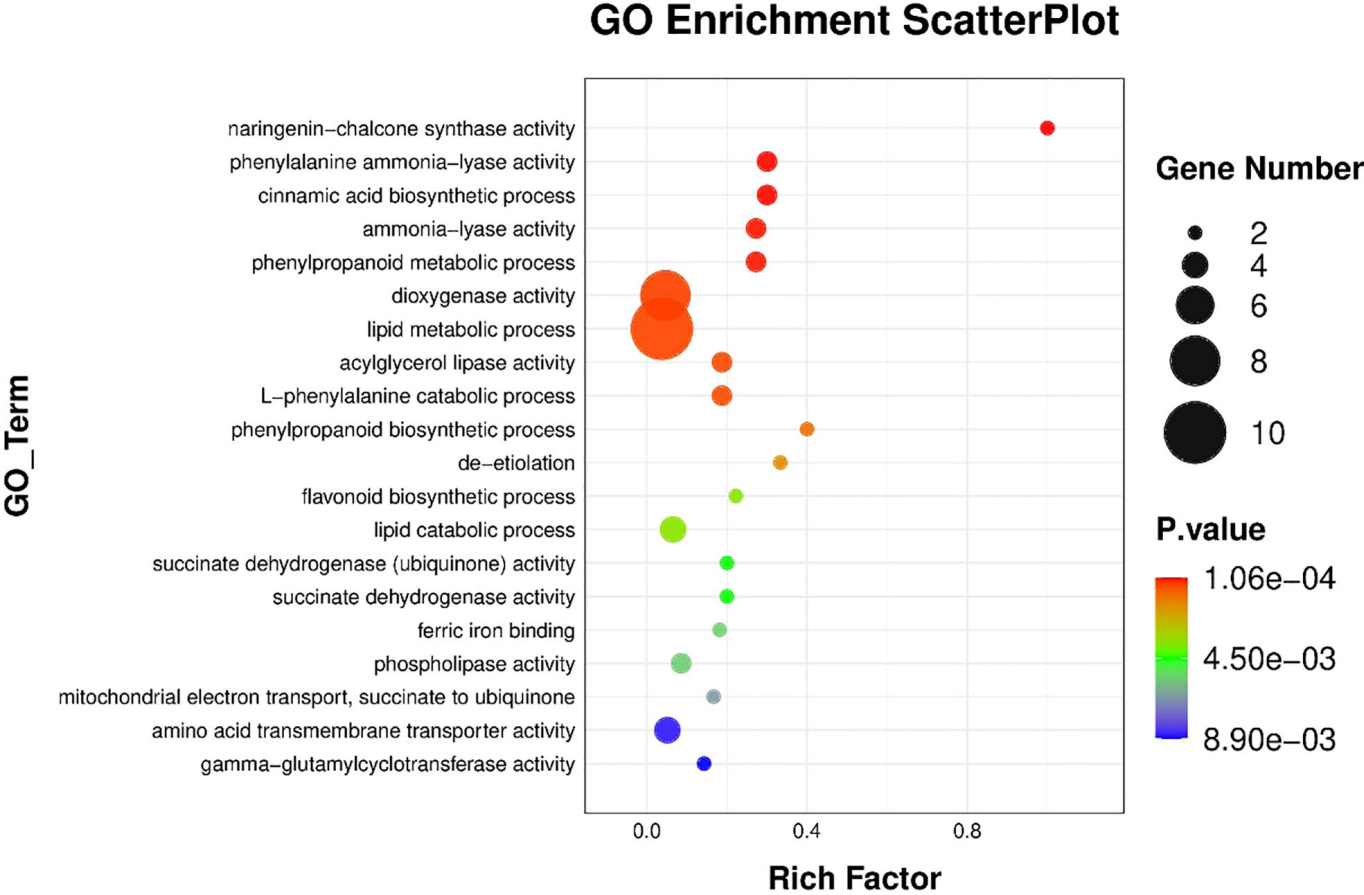
KEGG pathway enrichment scatter plot. The vertical axis represents the path name, and the horizontal axis represents the path factor corresponding to the rich factor.

**Figure 5:**
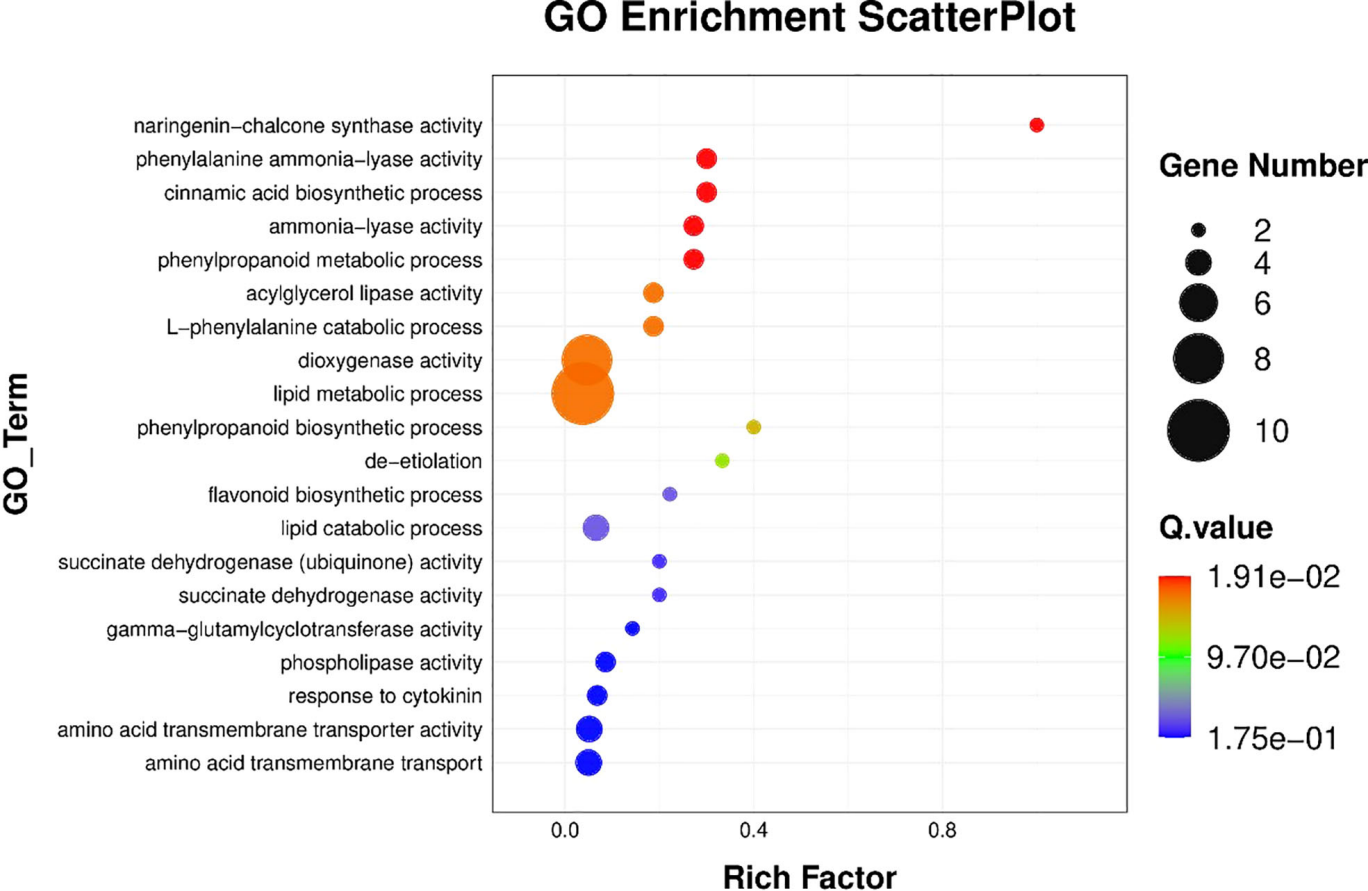
KEGG pathway enrichment scatter plot. The vertical axis represents the path name, and the horizontal axis represents the path factor corresponding to the rich factor.

**Figure 6:**
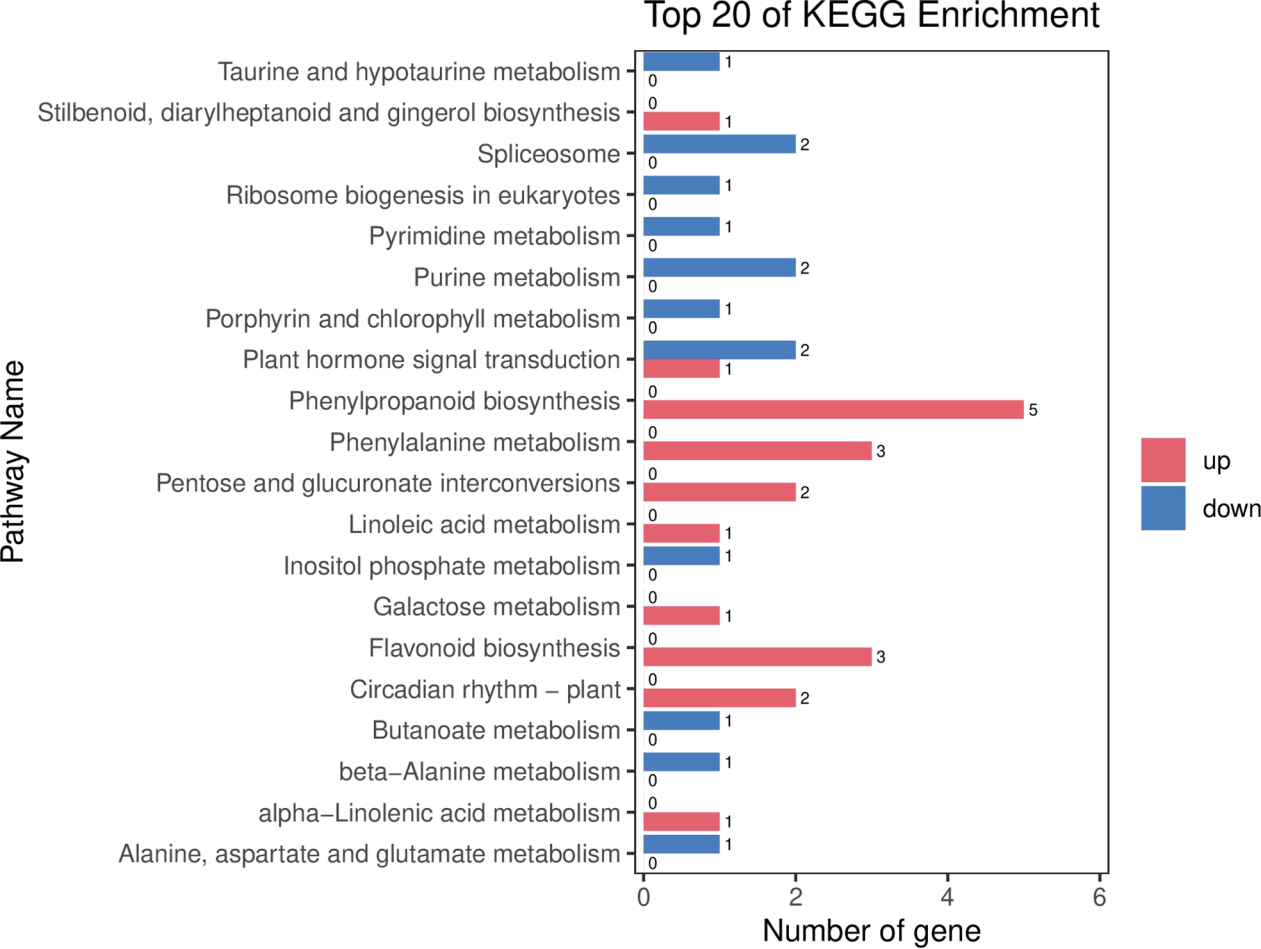
The differentially expressed genes or proteins are grouped hierarchically stretched GO terms, biological process, cellular components, and molecular functions.

The results of BRs were significantly different between the two genotypes, and occasionally, H21 displayed more BRs in contrast to 478 in its leaves. The DEGs were subjected to KEGG pathway improvement investigation in the systems of 2 versus 0 h, 16 versus 2 h, and 16 versus 0 h. The paths for plant height and root dry weight, chlorophyll content, number of leaves, shoot dry weight, and many more were improved in the DEGs with wider articulation. Numerous DEGs were improved into signal transduction pathways following the treatment for drought stress, including the calcium flagging pathway-plant and plant chemical signal transduction. Contrarily, under the drought treatment, only a small number of DEGs independently enhanced the KEGG pathway in drought-sensitive maize (**Figure 1**) and drought-tolerant maize (**Figure 2**). The x-hub shows improvement in KEGG pathway examination of DEGs of drought-sensitive (**Figure S1**) and drought-tolerant lines (**Figure 3**) under the drought treatment, individually (**Figure 4**). The x-hub shows an improvement score, while the y-hub demonstrates the pathway name (**Figure 5**). The size of the speck addresses the quantity of DEGs. The various shades of specks address different p-values (**Figure 6**).

### Transcriptome analysis for drought tolerance

**Figure 7:**
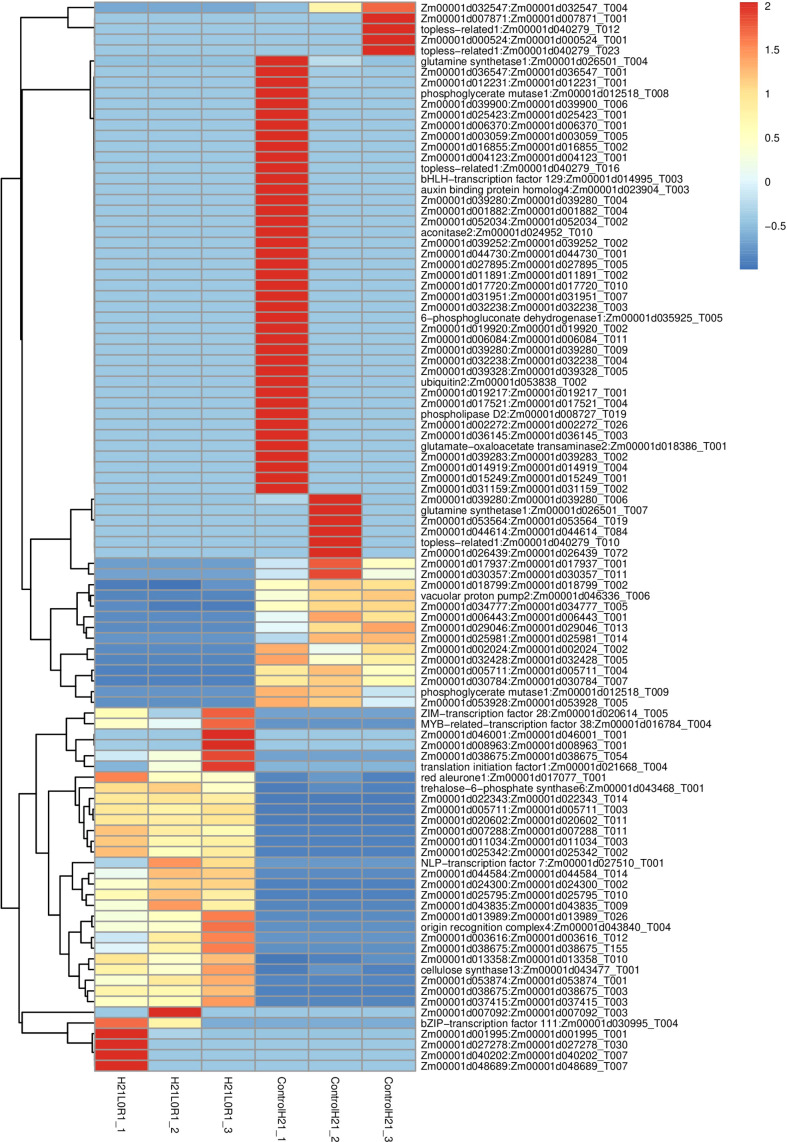
Transcriptomic profiling reveals shared signaling networks in different samples of maize.

The results obtained for normal conditions and drought or comparatively different as the sample show different results under normal conditions and similarly different under drought stress. The point at which they are linked showed that 18,184 rates divided among every treatment went from 83.86% to 90.21%. Two hundred nine characteristics could only be found in the drought-resistant cultivar (H21) after receiving drought treatment. H21 displayed a better plant height under drought stress conditions than 478. This cultivar (H21) had more apparent leaves than 478 (**Figure 7**). Even though the more mature leaves of 478 plants that were subjected to dry-season areas of strength showed signs of aging (the first two leaves were dry or yellow, and the third leaf additionally began to show signs of aging), this did not have any significant impact on H21, which consistently grew (just slightly toning down) significantly even when subjected to stress conditions.

Twelve cDNA libraries of the over two maize genotypes at the control and drought stress were built and sequenced with three organic duplicates to uncover the subatomic system of maize reaction to drought stress at the seedling stage. A sum of 20 G of clean information was obtained utilizing a reasonable stage. The complete base of each example was dispersed between 6.87 and 6.94 G, Q30 bases were circulated between 92.94% and 93.86%, and the typical GC content was 45.45%. By contrasting checks with the reference genome, the genomic arrangement of each example was obtained, and the arrangement rate was 96.25%–97.50% (**Figure 7**).

### PCA

**Figure 8:**
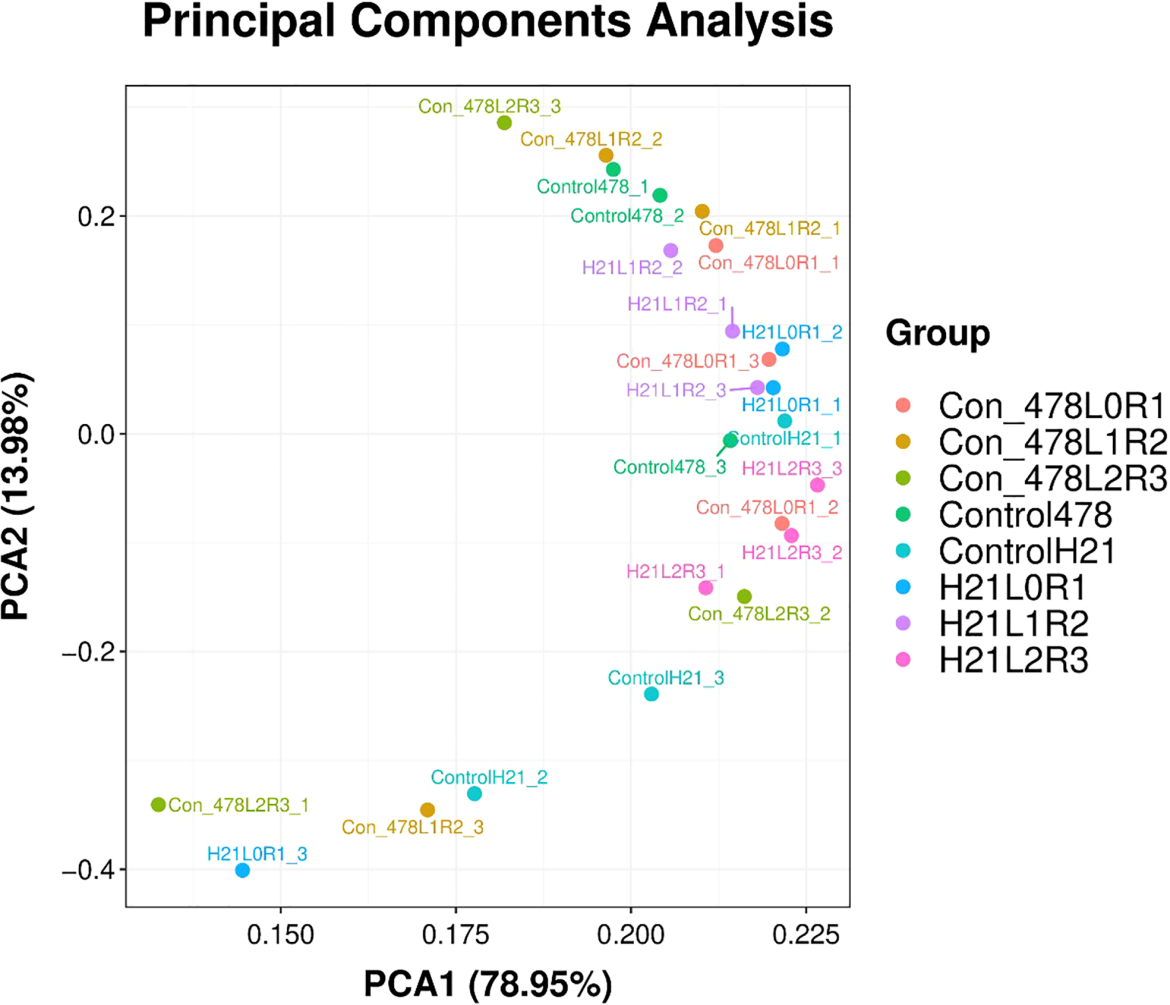
Categorization of different maize genotypes for drought tolerance and sensitivity. PCA with 78.95% values on the x-axis and 13.98% values on the y-axis.

It has been seen in **Figure 8** that PCA showed different values for H21 and different control values for 478, and the overall PCA value was found to be 13.98%, but on another note, they showed some values which are 4.87% for H21L0R1, 20.21% for control 478, and 10.67% for H21L2R3; similarly, values of 15.87%, 20.01%, 20.09%, 22.5%, and 17.5% for many other variables.

**Figure 9:**
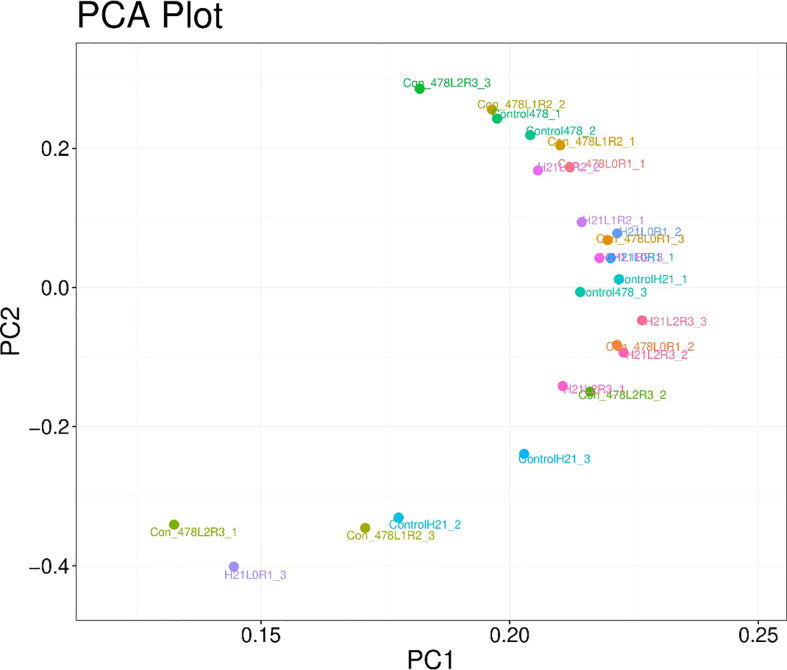
Categorization of different maize genotypes for drought tolerance and sensitivity. Principal component analysis of maize varieties based on different parameters visible in the form of colored spots with different values.

The number of H21 plants in stressful circumstances was much higher than in the control plants. This improvement was less notable in H21 than in 478. The 478 line exhibited greater peroxidation of membrane lipids based on the PCA content than in genotype H21, while lipid peroxidation appeared to rise more in H21 than in 478 following drought exposure (**Figure 9**).

The vital part examination has been displayed in (**Figure S2**), which shows that drought additionally decreased the effectiveness of the essential photosynthetic cycles. There were obvious contrasts between the control and drought-pushed plants, especially for the boundaries portraying plant height, chlorophyll content, etc. In any case, no obvious contrasts were traced between the genotypes for the various limitations of the PCA examination.

With drought stress, the distribution of excess energy was effectively extended (boundaries, plant height, and dry root weight). According to the extent of the red spots, which were concentrated on plants of one or the other genotype, the specific mass of the fourth leaf did not seem to be negatively impacted during the drought. The drought negatively influenced the availability of the individual plant units, as inferred from the blue areas on the figure, but the two genotypes did not differ. Our samples remained unaffected by drought for dry root weight H21. In any event, 478 demonstrated a more noticeable decline in the rate of chlorophyll content due to drought at the end of the chain because of the observed differences in the location of the specific orange spots (**Figure S3**)

Similarly, although transcriptome analyses are widely used, they can only provide a limited picture of substantial changes in value clarity. There is no need to be concerned about changes in plant cell transcriptome brought by drought reflecting in the cell proteome (because of many advances and factors affecting quality verbalization among RNA and protein levels); at the same time, it was evident that there was no undeniable connection between these two strategies for substantial value verbalization information, as the drought’s administration of alterations in plant proteome was segregated from the transcriptome’s advancements. For further evaluation, it may be helpful to examine the relationship between the transcriptional, translational, and posttranslational degrees of articulation of BR-related qualities in plants familiar with the dry period and their effects on the components of intracellular BRs and the overall plant response to this strain component.

### RT-PCR

**Figure 10:**
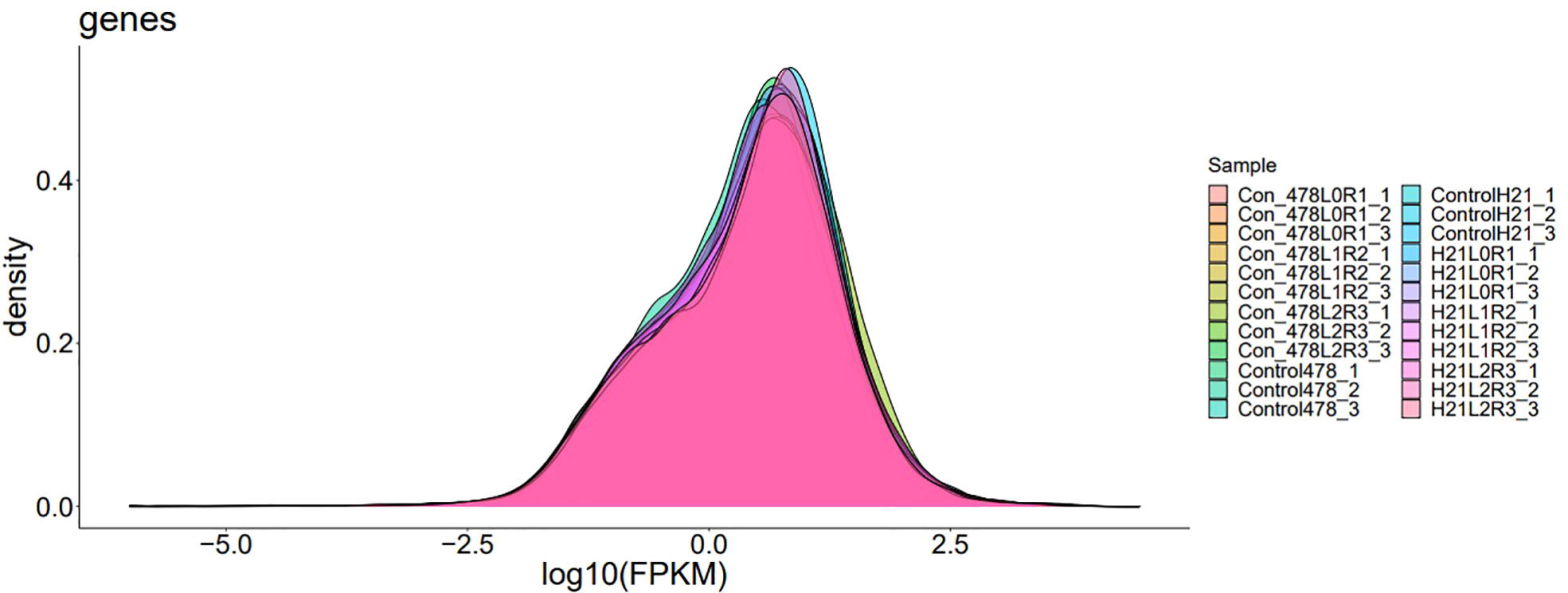
A variable peak with the second minor peak showing gene expression density; the gene density indicated the change in morphological parameters that were very clear during and after the whole analysis.

**Figure 11:**
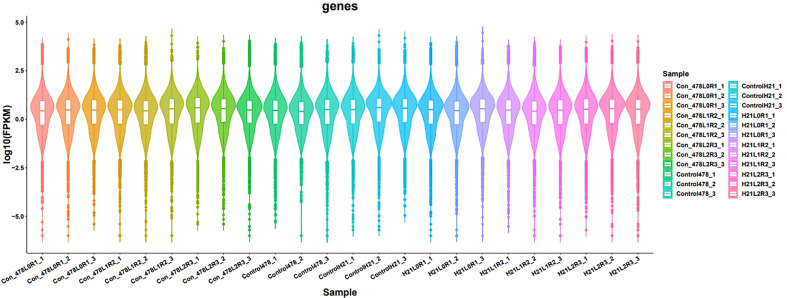
Results of different samples showing gene expression in the form of violin; the violin having different colors, indicating different samples analyzed at regular intervals.

**Figure 12:**
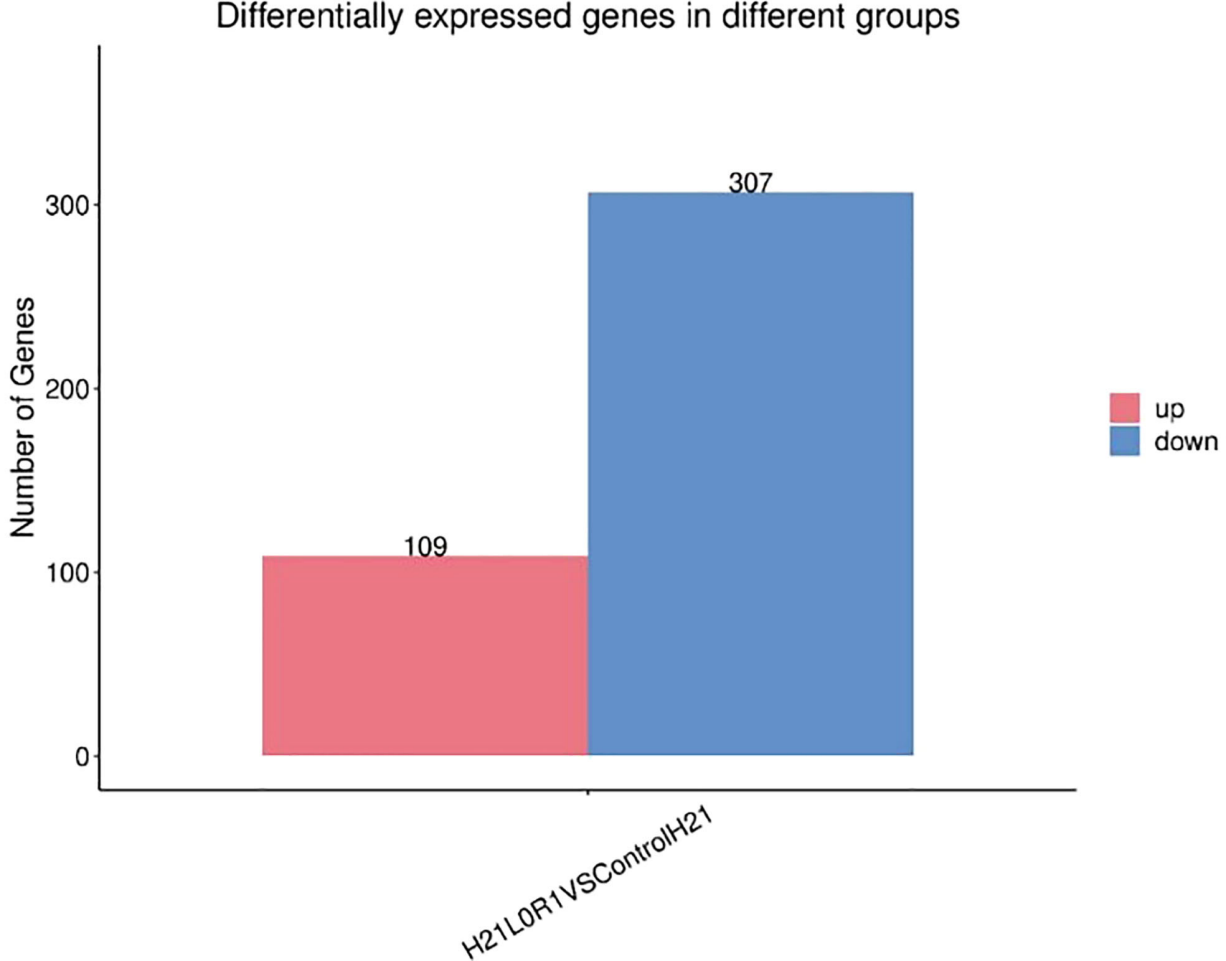
Differently expressed genes displayed as a bar graph in the different groups; the two bars showed higher and lower gene expression values in maize samples.

RT-PCR gave us information to identify differentially expressed genes that are mapped; reads of the genes were calculated using the sequence alignment/map (SAM) files created by Tophat2. Then, differential expression analyses between two groups of samples were performed using the R package “DESeq2.” DEGs can be considered the best method for a graphical or visual representation of RT-PCR. Genes with |log2 (fold changes) |>1 and false discovery rate (FDR) <0.02 were recognized as differentially expressed. To detect the DEGs, four comparison groups, i.e., LMC_LMD (Lv28 under moderate drought control vs. Lv28 under moderate drought), LSC_LSD (Lv28 under severe drought control vs. Lv28 under severe drought), HMC_HMD (H21 under moderate drought control vs. 478 under moderate drought), and HSC_HSD (H21 under severe drought control vs. 478 under severe drought), were included as shown in **Figure 10**. Differential expression analysis was performed by comparing the gene expression profiles between different drought conditions to identify genes responding to drought in the two lines. In total, 100% and 51% of genes showed drought response under MD and SD in 478, respectively, among which 28% and 26% were upregulated under MD and SD, while 16% and 12% of genes were downregulated, respectively (**Figure 11**). In the drought-tolerant line H21, 68% and 33% of genes showed response to drought stress under MD and SD, respectively, among which 62% and 59% genes were upregulated, while 16% and 15% genes were downregulated, respectively (**Figure 12**). Remarkably, the number of the DEGs increased in 478 from MD to SD while it decreased in Lv28. In addition, the results of RT-PCR displayed as DEGs in H21 showed intensively upregulated under MD, with 82% genes upregulated but only 32% genes downregulated. Among these DEGs, 84% of genes responded to drought in H21 and 478 (**Figure S4**).

## Discussion

In the current work, we have presented comprehensive details of maize’s physiological and transcriptomic data under drought stress. Under simulated drought stress, it may be generalized that maize demonstrates stress tolerance mechanisms. The levels of activity arise following stress treatment, along with conductivity and soluble sugar content, suggesting that maize had improved scavenging abilities to withstand drought (**Table 1**). It was displayed that a rise in enzyme activity might remove peroxides produced by stress, preventing the plasma membrane’s oxidation and safeguarding cells against injury. According to our findings, maize controls the activity of defense enzymes, to reduce the accumulation of harmful substances (Zenda et al., 2018).

Additionally, we have also performed many analyses that gave us graphical results (Muthurajan et al., 2018). The results were incredibly significant and demonstrated that hormones could reduce growth under drought stress (Chen G. et al., 2019). Moreover, although transcriptome analyses are widely used, they only provide a very restricted result that causes changes (**Table 3**). According to the results of the KEEG improvement analysis, a total of the top 20 results were improved in the BRs of H21 and 478, including 15 in the cosmology of “organic cycle,” nine in the philosophy of “sub-atomic capacity,” and seven in the metaphysics of “cell component (Tiwari et al., 2021).” The DEGs of H21 and 478 had several advanced results, including eight organic cycle terms affecting the stress response (Chen G. et al., 2019).

**Table 3:** Morphological and physiological trait analysis.

Sr. #	Traits	Mean	CV	Skewness	Kurtosis	Min	Max	Control values	
								Min	Max
1	Plant height	14.04 ± 9.45	11.98	−0.77 **	0.38	20.0	10	15	18
2	Root dry weight	20.14 ± 5.08	11.22	0.63 **	0.05	6.2	15.8	13	17
3	Shoot dry weight	19.38 ± 8.75	18.46	0.50 **	−0.05	7.5	14.1	10	15
4	Number of leaves	0.58 ± 0.14	11.43	2.99 **	16.85 **	0.2	2.5	5	8
5	Specific mass of the fourth leaf	11.39 ± 16.18	14.53	1.07 **	1.29 **	6.7	10	7	10
6	A.O activity	12.40 ± 11.64	12.15	1.77 **	4.98 **	3.3	16.7	6	8
7	CAT	1.81 ± 0.44	12.16	1.54 **	3.06 **	0.7	5.2	13	16
8	MDA	17.95 ± 12.183	19.14	−0.02	−0.66 *	0	19	11	14
9	Chlorophyll content	2.14 ± 1.343	18.73	1.34 **	1.76 **	0	11.6	15	19

*Significant.

**Highly significant.

At the same time, if we talk about the results of our samples, i.e., H21 and 478, they did not show similar changes that were related to KEEG results (Xuan et al., 2022). Furthermore, all four sets of BRs showed that the “extracellular region” and “starch metabolic cycle,” showing two lines, included similar drought blockage components (Zenda et al., 2018). PCA revealed the identical characteristics of both plants, including the relevance of numerous limitations (Estrella-Maldonado et al., 2021). Each rate is shown with the eigenvalues in the PCA evaluation, indicating that these characteristics are accurate and connected to the other rates (**Table 3**).

Drought significantly reduced the growth of maize lines. We used seedlings of the maize line under controlled and drought conditions for a time-series transcriptomics study to identify DEGs and their patterns of response to increasing drought imposition (Zenda et al., 2019). In conclusion, drought stress inhibited enzyme activity linked to the production of cellular components, which is consistent with the suppression of plant growth (Yang et al., 2015). On the other hand, stress resistance mechanisms and regulatory activities are enhanced. We can evaluate dynamic gene responses to steadily rising drought stress using time-series transcriptomic data (Zeng et al., 2019). The samples, significance to drought tolerance, and interactions with sRNAs are other ways to define DEGs (Ghodke et al., 2020).

These results showed that the drought resistance of H21 may be increased by the root development, stability of the cytoskeleton, and performance of various cell cycles during drought. According to the KEGG enhancement analysis, 10 metabolic pathways were enhanced in the two lines (Wang et al., 2019). Starch and sucrose digestion, as well as plant chemical signal transduction, were fully developed in both lines. Still, their levels in H21L0R3 were typically greater than those in 478. Many researchers who worked with exogenous BR samples, transgenic plants, and earlier plants that had been kept with BRs before being exposed to lack of circumstances investigated the heights of numerous express records that were linked with drought reaction (**Table 3**).

*Arabidopsis thaliana*, *Brassica napus*, *Cucumis sativus*, *Nicotiana tabacum*, *Solanum tuberosum*, *Brachypodium*, or grains were the subjects of these tests (Ghodke et al., 2020). Even while the levels of CPD recordings dynamically increased over the first 24 h of PEG-induced osmotic stress in potatoes, it has not been thoroughly investigated whether drought-stressed plants exhibit increased BR biosynthetic properties or not (Wang et al., 2016). The early C-22 oxidation pathway and the late C-6 oxidation pathway both include several catalyzed stages (Qiu et al., 2019). In maize coding for H21, supplementary BR biosynthetic quality was dispersed.

This rate-limiting protein catalyzes different hydroxylation reactions at the beginning of early and late C-6 corrosion trials and the first H21. A vast phenotypic variability was discovered for all traits to handle the circumstances and start a dry phase (He et al., 2020). Chlorophyll content and the dry load decreased by 90% during the dry spell, while plant height decreased by 40%. Except for plant height, the variety ranges were smaller under drought stress compared to control circumstances, and the base was zero for all characteristics (Wang et al., 2015). Information obtained indicated that the properties were transported in a symmetrical manner (Tang et al., 2017).

In addition to identifying genes that may be involved in drought tolerance and other abiotic stresses, our research provides extensive transcriptome data enabling comparisons with gene responses to drought in other tissues under various drought circumstances (Zhang et al., 2017). Our results show that early and middle DEGs have greater genetic diversity and are more likely to be associated with drought resistance (Hu et al., 2018). The role of early and middle DEGs in drought tolerance involves huge levels of genetic variability. They include the gain and loss of gene function that may emerge from maize lines’ adaptability to changing growth circumstances (Jin et al., 2019).

A type of CNV called presence and absence variation (PAV) results from the lack of stress-responsive genes in the pathways that respond to environmental stressors under specific circumstances to reduce fitness costs. The discovery corroborates this that several crop species, including maize, soybean, and grape, have genes with CNV overrepresentation in the pathways of stress responses (Thirunavukkarasu et al., 2017).

Under drought circumstances, genetically speaking, the tolerant genotype H21 showed greater levels of metabolites, lower levels of lipid peroxidation, and better cell water retention than the sensitive genotype 478. In all, sample genomes analyzed with the help of RT-PCR in the form of DEGs were identified in our RNA-seq data as being expressed in response to drought (Zhang et al., 2019), with many DEGs being specifically discovered in H21. We discovered that genes linked to previously described pathways implicated in the drought stress response, such as those linked to the production of secondary metabolites, transcription factor regulation, detoxification, and stress defense, were changed upon exposure to drought stress.

The contribution of H21's characteristics associated with cell wall biosynthesis to the water maintenance of cell wall mix has regions of strength thanks to the input of a coordinated multienzyme complex in polysaccharide production. The raised articulation of glycosyltransferase added to the drought barrier of *Arabidopsis* thaliana cytokinin-inadequate freaks (Zenda et al., 2018). GO advancement examination uncovered that numerous H21 DEGs were connected with cell wall association and RT-PCR, particularly the expansion family prompted by different abiotic stresses and ABA. Stomatal thickness was displayed to diminish by overexpression. Furthermore, RT-PCR functional validation analysis supported the discovered genes’ differential expression. Our discoveries not only increase our understanding of the mechanisms that allow maize to withstand drought stress but also offer a priceless genetic resource or selection target for maize’s genetic advancement (Yang et al., 2015).

Additionally, GO and KEGG enrichment analyses on DEGs showed that several differentially expressed genes were most strongly enriched in biological processes, such as citrate cycle (TCA cycle), fatty acid metabolism, carbon fixation in photosynthetic organisms, and ribosome, enzymes distributed with a high proportion (Lei et al., 2022), indicating that a variety of metabolites were synthesized *via* active metabolic processes in leaves of maize. The RNA-seq technique can potentially identify novel genes from non-model organisms.

These observations have also provided a helpful resource for studying specific processes and pathways involved in growth and development of various plants and other plant species. The pathway-based analysis helps in comprehending the traits and subsequent real cycles. Maize has economic and conventional significance due to its distinct model and unique germplasm (Ereful et al., 2020). Additionally, it serves as a model plant for research on regulatory interactions. The investigation of maize drought tolerance has produced incredible advances in understanding this complex trait (Xia et al., 2020).

The results obtained after the analysis will help further research in the same area related to drought or any other similar plant that showed the same mechanism. Finding genes from maize that are connected to drought resistance and using them to increase the yield in various drought environments becomes a crucial breeding goal. It is theoretically and practically very important to analyze maize’s mechanism for drought tolerance (Vojta et al., 2016). Numerous regulatory mechanisms, including increased proline, soluble sugar, malondialdehyde, superoxide dismutase, potential operational delineations, and catchment area treatment content buildup were evolved in maize to deal with drought and to prevent or limit cellular damage induced by drought stress. Improved metabolic pathways were identified by pathway improvement assessment in distinct DEGs and the establishment of the entire genome. Omic Share was used for pathway headway evaluation to identify drastically altered metabolic or signal transduction pathways in several features (Tarun et al., 2020). Our study showed that applications of Br hormone could reduce the toxic effects in maize in inbred lines Br application increased the gene expressions and increased the drought tolerance in maize inbred lines.

## Conclusions

The transcriptome analysis uncovered the differential expression of genes controlling drought tolerance in maize. Two contrasting maize genotypes performed differently under drought stress conditions. Drought stress leads to noticeable changes in maize genotypes. Results indicated that the drought-responsive maize genotype performed well under drought stress and as indicated by a differentially expressed gene network. DEGs are involved in different stress-responsive pathways and control several vital traits under drought stress. BRs played a key role in promoting plant growth under drought-stress conditions. The application of BRs improved the growth of maize genotypes and activated several stress-responsive genes. BRs compensated for the drought-induced toxic changes in maize genotypes and improved root growth, shoot growth, and chlorophyll contents. Using maize lines with reciprocal drought resistance, transcriptomes were examined under dry periods to identify several BRs. The BRs linked with plant chemical sign transduction, osmotic change, root advancement, and receptive oxygen rummaging were enhanced under drought lenient lines. Our outcomes showed drought-responsive components and gave new insights into understanding drought resistance in maize.

## Data availability statement

The raw data supporting the conclusions of this article will be made available by the authors, without undue reservation.

## Author contributions

SG and YP contributed to the study concept and design and statistical analysis. SG, AA, IU, and ZZ contributed to the analysis and interpretation of data. YP contributed to the investigation and resources. SG and SA contributed to the drafting of the manuscript. MK, AR, RS, and YW contributed to the review, editing, and proofreading of this manuscript. YP contributed to funding acquisition and study supervision. All authors contributed to the article and approved the submitted version.

## Funding

This research was supported by the industrial support plan for colleges and universities of Gansu,China (No. 2022CYZC-46), the Fuxi Talent Project of Gansu Agricultural University, China (No. GAUFX-47102Y09), and the Lanzhou Science and Technology Bureau (No. 2020-RC-122).

## Conflict of interest

The author Dr. Adnan Rasheed is employed by Jilin Changfa Modern Agricultural Science and Technology Group Co., Ltd.

The remaining authors declare that the research was conducted in the absence of any commercial or financial relationships that could be constructed as a potential conflict of interest.

## Publisher’s note

All claims expressed in this article are solely those of the authors and do not necessarily represent those of their affiliated organizations, or those of the publisher, the editors and the reviewers. Any product that may be evaluated in this article, or claim that may be made by its manufacturer, is not guaranteed or endorsed by the publisher.

## Supplementary material

Supplementary Figure 1 Venn diagram showing the relationship between the differentially expressed genes. The values in each circle represent the number of genes.

Supplementary Figure 2 Principal Component Analysis (PCA) shows results using a scattered graph with 54.46% variance on the x-axis and 14.29% on the y-axis.

Supplementary Figure 3 The expression numbers of transcriptomic responses in each treatment using a PCA, three peak lines showing variations in the analysis values.

Supplementary Figure 4 Differently expressed genes displayed as Bars graph in different groups two bars showed gene expression one having a higher number of transcripts and other with lower.

## Abbreviations

ANOVA, Analysis of variance; PCA, Principal component analysis; Q-PCR, Quantitative polymerase chain reaction; BRs, Brassinosteroids; KEGG, Kyoto Encyclopedia of Genes and Genomes; SOD, Superoxide dismutase; POD, Peroxidase; CAT, Catalase.
