# Supplementary figures and images for: Brassinosteroids induced drought resistance of contrasting drought-responsive genotypes of maize at physiological and transcriptomic levels
Source: Front Plant Sci. 2022 Oct 25;13:961680. doi: 10.3389/fpls.2022.961680 (PMC9641234; doi:10.3389/fpls.2022.961680)

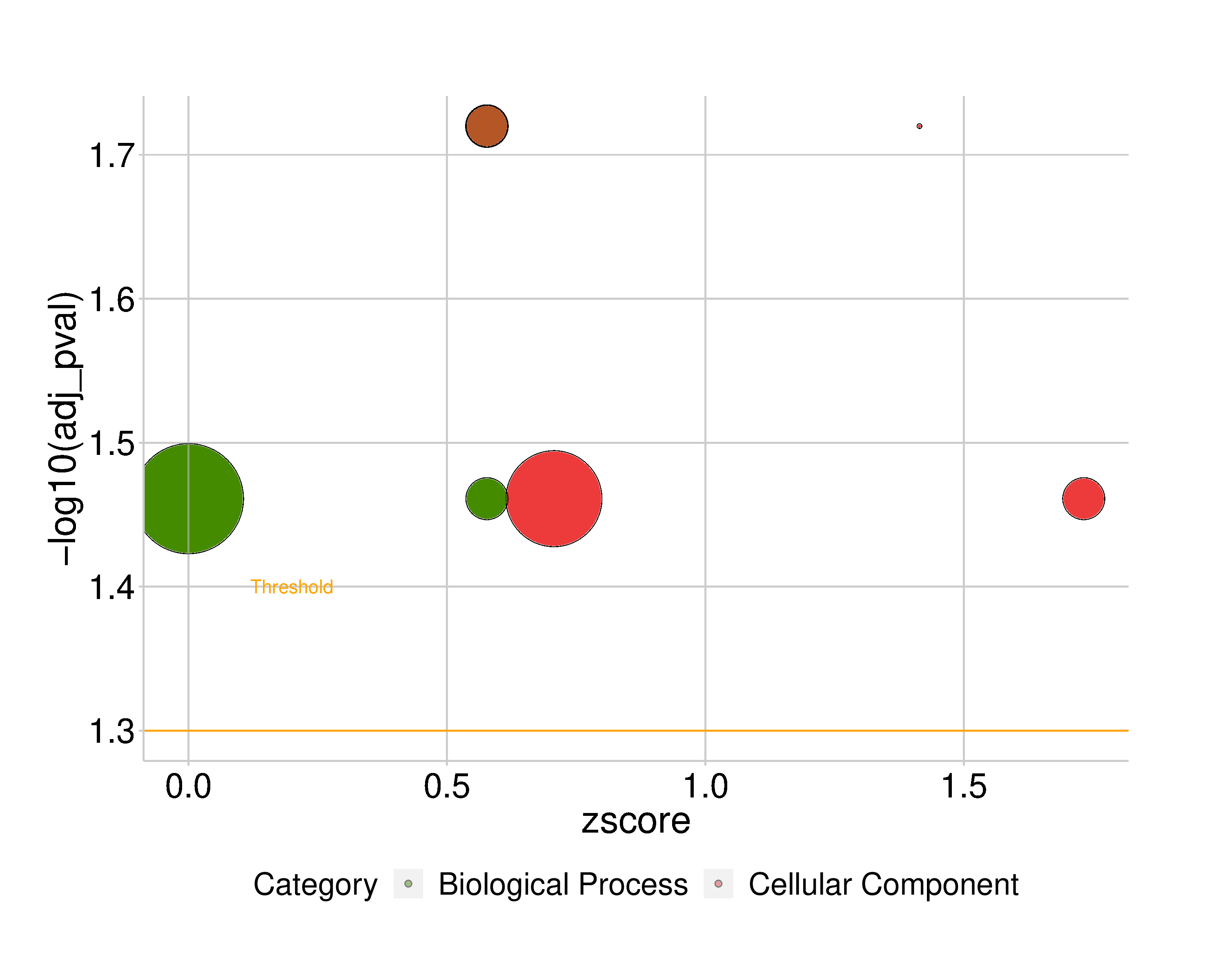

Supplement: Supplementary Figure 1 — Venn diagram showing the relationship between the differentially expressed genes. The values in each circle represent the number of genes. [file Image_1.png]

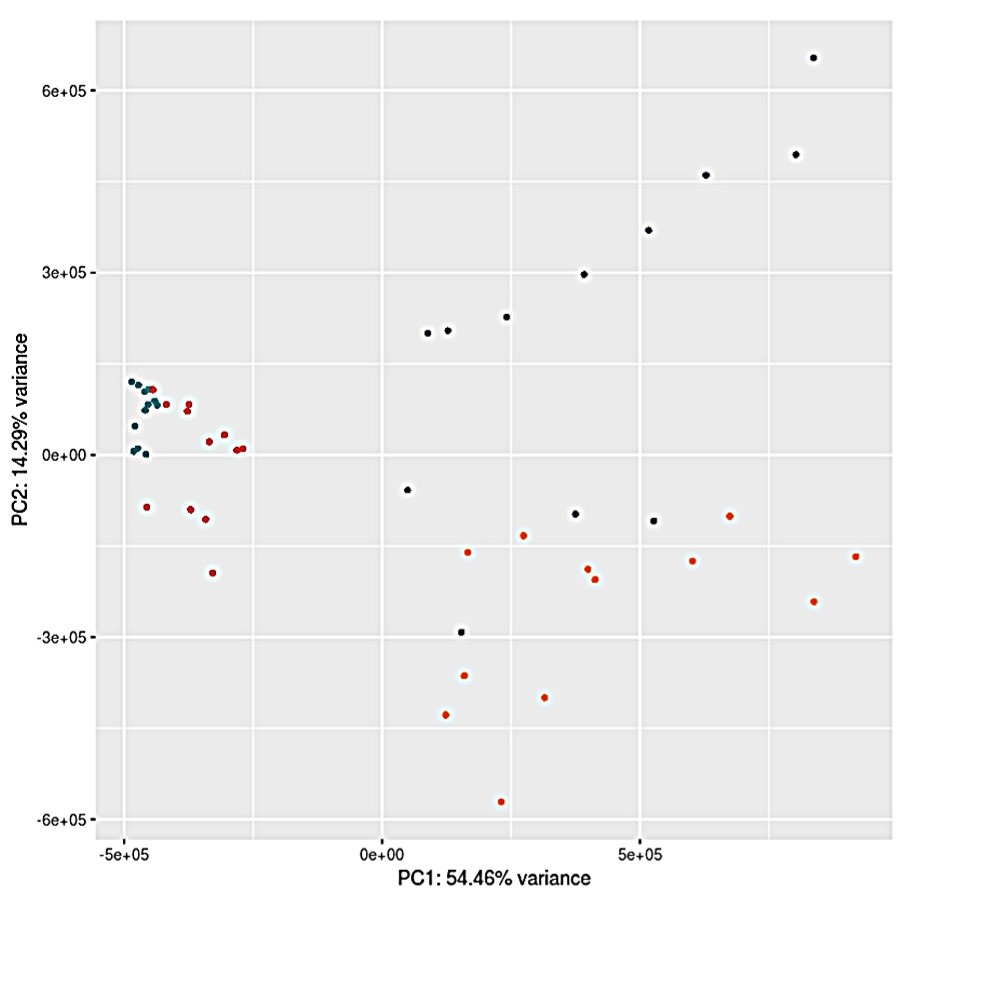

Supplement: Supplementary Figure 2 — Principal Component Analysis (PCA) shows results using a scattered graph with 54.46% variance on the x-axis and 14.29% on the y-axis. [file Image_2.jpeg]

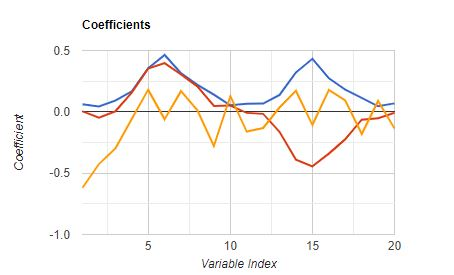

Supplement: Supplementary Figure 3 — The expression numbers of transcriptomic responses in each treatment using a PCA, three peak lines showing variations in the analysis values. [file Image_3.tif]

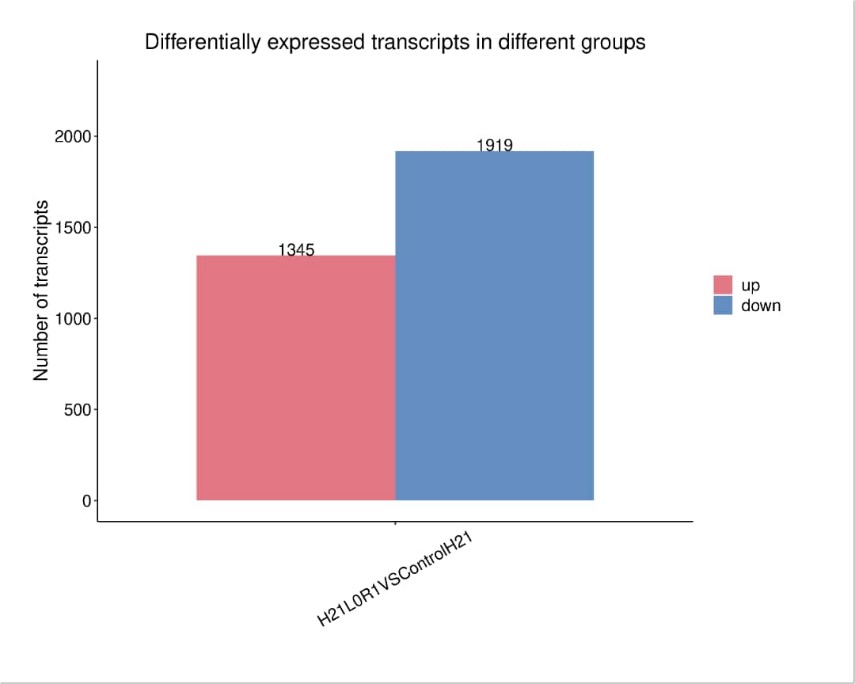

Supplement: Supplementary Figure 4 — Differently expressed genes displayed as Bars graph in different groups two bars showed gene expression one having a higher number of transcripts and other with lower. [file Image_4.jpeg]
